# Effect of Aging and Glucagon-like Peptide 2 on Intestinal Microbiota in SD Rats

**DOI:** 10.14336/AD.2017.1001

**Published:** 2018-08-01

**Authors:** Jiayu Wu, Weiying Ren, Li Li, Man Luo, Kan Xu, Jiping Shen, Jia Wang, Guilin Chang, Yi Lu, Yiming Qi, Binger Xu, Yuting He, Yu Hu

**Affiliations:** ^1^Department of Geriatrics, Zhongshan Hospital, Fudan University, Shanghai 200032, China; ^2^Department of Internal Medicine, Zhongshan Hospital, Fudan University, Shanghai 200032, China

**Keywords:** aging, GLP-2, intestinal microbiota, high-throughput sequencing

## Abstract

Recent research suggests that intestinal microbiota affect the aging process. Glucagon-like peptide 2 (GLP-2), a growth factor found in the intestinal mucosal epithelium, reduces intestinal permeability and affects intestinal microbiota. The relationship between aging, GLP-2, and intestinal microbiota are still not well understood. The current study examined the influence of aging and GLP-2 on the intestinal microbiota of rats. Twelve 3-month old male SD rats were randomly divided into two groups: a young control group (group C) and a young GLP-2 treatment group (group G). Twelve 26-month old male SD rats were randomly divided into two groups: an aged control group (group L) and an aged GLP-2 treatment group (group T). GLP-2 was intraperitoneally injected into rats from group G and group T for 14 days. Plasma GLP-2 concentration was evaluated by ELISA tests. Fresh intestinal stool samples were collected from each group and total fecal bacterial genomic DNA was extracted from the associated rats. The bacterial composition of fecal samples was analyzed by Miseq high-throughput sequencing and comparison with SRA databases. Overall, the diversity of intestinal microbiota significantly decreases with age in SD rats, while GLP-2 has no significant effect on the diversity of intestinal microbiota. Upon aging, there is a reduction in probiotic bacteria and a concomitant increase in pathogenic bacteria in rats. Treatment with GLP-2 results in a significant reduction in the prevalence of pathogenic bacterial genera and an increase in some potential benefit bacteria in aged rats. In addition, treatment with GLP-2 results in an increase in several probiotics and a reduction in the prevalence of pathogenic bacterial genera in young rats.

Intestinal microbiotas are not only composed of parasitic microorganisms, but also of symbiotic bacterial species that affect the health and coevolution of humans and animals [1]. The composition of the symbiotic bacterial populations that inhabit the gastrointestinal tract is extremely diverse (up to 500-1000 species). Furthermore, there are approximately 100 trillion bacterial cells in the gastrointestinal tract, which is over 10 times the number of host cells [2]. The number of genes encoded by members of the intestinal microbiota is approximately 150 times that encoded by the host [3]. It is well known that the intestinal microbiota plays an important role in host nutrition, glucose and lipid metabolism, anti-inflammation and immune regulation [4]. In recent years, it has also been suggested that the composition of the microbiota also affects the process of aging [5]. Some studies have shown that there is a reduction in *Bacteroidetes*/*Firmicutes* (B/F) in the intestinal microbiota of aging rats. Furthermore, the diversity of the intestinal microbiota of aging rats is also reduced [6]. Other studies have revealed that aging causes an increase in the serum concentration of LPS, IL-6, IL-2, IL-1β and TNF-α and a decrease in the serum concentration of IL-10 [7]. It is speculated that the latter occurrence is caused by an intestinal microbiota imbalance that is promoted by intestinal barrier imbalances and the upregulation of chronic low-grade inflammation. However, in most of the studies that analyzed the latter phenomenon, models that simulated aging were generated following the administration of drugs [8]. Hence, it is likely that these models did not accurately reflect changes in the intestinal microbiota experienced by naturally aging rats.

Glucagon-like peptide 2, a specific growth factor found in the intestinal mucosal epithelium, can promote intestinal growth and nutrient absorption. This protein is also involved in reducing intestinal permeability, maintaining the intestinal barrier function and repairing injuries associated with intestinal diseases [9, 10]. Furthermore, Cani PD et al. reported a correlation between intestinal microbiota and GLP-2 following the administration of probiotics to obese mice. The same study confirmed that *Bifidobacterium* could increase the production of endogenous GLP-2 in mice [11]. A separate study by the same authors revealed similar results in obese rats [12]. In recent years, additional studies have confirmed that probiotics can increase the production of endogenous GLP-2 in healthy populations [13] and glucose-tolerant humans [14]. It has also been suggested that GLP-2 can influence the composition of the intestinal microbiota due to its role in nutrient absorption and barrier protection in the intestinal tract. If GLP-2 has an effect on the intestinal microbiota, treatment with GLP-2 may be useful for improving the change in intestinal microbiota caused by aging.

In order to study the influence of aging and GLP-2 on the intestinal microbiota of rats, aging rats were utilized in this study. Following administration with GLP-2, changes to the diversity and relative abundance of the intestinal microbiota of rats were monitored using Miseq high throughput sequencing. This experiment was performed to preliminarily explore the relationship between aging, GLP-2 and the intestinal microbiota.

## MATERIALS AND METHODS

### Animals and sample collection

Twelve 3-month-old male SPF SD rats and twelve 26-month-old male SPF SD rats were used in this study. All animal care procedures were approved by the Laboratory Animal Center of Fudan University, Shanghai Medical College and the Animal Studies Committee of Fudan University, China. All of the rats were housed together in groups of six in a specific pathogen-free standard animal laboratory (with a 12 h: 12 h light/dark cycle, 22-25°C ambient temperature and 45-70% relative humidity) and fed ad libitum with standard chow for 1 week prior to treatment initiation. Commercial rat chow (SLAC; Shanghai, China) and water were autoclaved prior to use.

The 3-month old rats were randomly divided into 2 groups with 6 rats per group. The 26-month-old rats were also randomly divided into 2 groups with 6 rats each:

Group C: Rats aged 3 months were injected intraperitoneally with normal saline twice a day for 14 days.

Group G: Rats aged 3 months were injected intraperitoneally with GLP-2 (250 μg·kg^-1^·d^-1^) (APC; Rhode Island, USA) twice a day for 14 days.

Group L: Rats aged 26 months were injected intraperitoneally with normal saline twice a day for 14 days.

Group T: Rats aged 26 months were injected intraperitoneally with GLP-2 (250 μg·kg^-1^·d^-1^) twice a day for 14 days.

All of the treated rats were housed, fed and watered in an identical manner prior to and after treatment initiation.

The rats were euthanized prior to extraction of fecal samples from the terminal ileum and collection of blood by direct cardiac puncture on the 15^th^ day of treatment. The fecal samples (24 in total) were immediately frozen in liquid nitrogen. After the fecal samples were thoroughly frozen, they were stored at -80°C until DNA was extracted. Blood samples were collected in tubes with EDTA plus Sigma diprotin. The blood samples were centrifuged at 3000 × g for 15 min at 4°C and the separated plasma was stored in a freezer at -80 °C for later analysis of GLP-2.

### DNA extraction, PCR amplification and Miseq sequencing

Total genomic DNA from each sample (100 mg) was extracted using the E.Z.N.A. DNA stool purification kit (Omega Bio-tek Norcross; GA, USA) according to the manufacturer’s instructions. The V4-V5 region of the bacterial 16S ribosomal RNA gene was amplified by PCR (95°C for 2 min, followed by 25 cycles of 95°C for 30 s, 55°C for 30 s, and 72°C for 30 s and a final extension step at 72°C for 5 min) using the following primers (SBC; Shanghai, China): 338F, 5’-barcode-ACTCCTACGG GAGGCAGCA-3’ and 806R, 5’-GGACTACHVGGG TWTCTAAT-3’, where the barcode represents an eight-base sequence unique to each sample. PCR reactions were performed in triplicate in a 20 μl final volume containing 4 μl of 5 × FastPfu Buffer (SGMB; Shanghai, China), 2 μl of 2.5 mM dNTPs and 0.8 μl of 5 μM sequence-specific primers. PCR reactions were performed using 10 ng of template DNA.

Amplicons were extracted from 2% agarose gels (Bio Rad; CA, USA) and purified using a DNA Gel Extraction Kit (AxyGen; CA, USA) according to the manufacturer’s instructions. The resultant gel extracts were quantified using QuantiFluor™-ST (Promega Corporation; Madison, USA). Purified amplicons were pooled in equimolar amounts and paired-end sequenced (2 × 250 bp reads) using an Illumina Miseq high-throughput PE 300 Sequencing Platform (Illumina, USA) in accordance with standard protocols. The raw reads were deposited into the NCBI Sequence Read Archive (SRA) database.

Raw fast files were demultiplexed and quality-filtered using QIIME (version 1.9.1) according to the following criteria: (i) the 300 base pairs reads were truncated at any site receiving an average quality score of less than 20 over a 50 base pair (bp) sliding window and truncated reads that were shorter than 50 bp were discarded, (ii) exact barcode matches, reads containing 2 nucleotide mismatches following primer matching or reads containing ambiguous characters were removed, and (iii) only sequences containing overlaps that were longer than 10 base pairs were assembled according to their overlap sequence. Reads which could not be assembled were discarded.

### Bioinformatic analysis

Operational Taxonomic Units (OTUs) were clustered with 97% similarity cutoff using UPARSE (version 7.1, http://drive5.com/uparse/) and chimeric sequences were identified and removed using UCHIME. Taxonomical classification of each 16S rRNA gene sequence was performed using RDP Classifier (http://rdp.cme.msu.edu/) and the SILVA (SSU123) 16S rRNA database using a confidence threshold of 70%.

The OTUs that reached at a 97% nucleotide similarity level were used for alpha diversity (Shannon, Simpson, and Coverage), richness (ACE and Chao), and rarefaction curve analyses. Phylogenetic beta diversity measures such as unweighted UniFrac principal coordinate analysis (PCoA) were performed using OTUs for each sample [15]. The RDP classifier Bayesian algorithm for 97% similarity levels of OTUs was used for representative sequences during phylum and genus taxonomic analysis.

### Plasma analysis

Plasma samples of 10 ul were added to a 1.5 ml tube of polypropylene containing 450 ul sample diluent. The plasma was acidified with 1 N hydrogen chloride at 4°C and placed for 60 min. Then the tube was added with 1 N sodium hydroxide and blended. The plasma levels of GLP-2 were measured by ABC ELISA kits (Xinqidi Bio, Wuhan, China) according to the manufacturer’s instructions.

### Statistical analysis

The Kruskal-Wallis sum-rank test, Mann-Whitney U test, ANOVA analysis and Pearson correlation coefficients were performed using SPSS version 17.0 for Windows. All of the optimized OTU sequences that were similar to the OTU sequences from selected representatives (greater than 97% similarity) were produced in the OTU forms.

## RESULTS

### Diversity of the gut microbiota in rats

Miseq sequencing resulted in 226,798,684 raw sequences including 127,924,964 high-quality raw sequences with a median read length of 438.13 base pairs (ranging from 401 to 500 base pairs). Repetitive sequences were extracted using the Usearch platform and non-repetitive sequences were clustered into OTUs at 97% similarity.

**Table 1:** Diversity estimation of the intestinal microbiota in SD rats.

Group	No. of reads	No. of OTUs	Richness estimator				α-Diversity index			

			Ace	95% CI	Chao	95% CI	Coverage	Shannon
**Group C**	69927	2124	413	393.1-431.9	415	384.9-445.8	0.9893	4.40
**Group G**	72390	2111	400	386.5-414.1	406	392.3-419.7	0.9909	4.55
**Group L**	78031	1848	367	345.3-388.4	372	336.0-408.0	0.9916	3.90
**Group T**	71638	1912	371	339.0-402.7	385	345.7-424.3	0.9910	3.82

**Figure 1:**
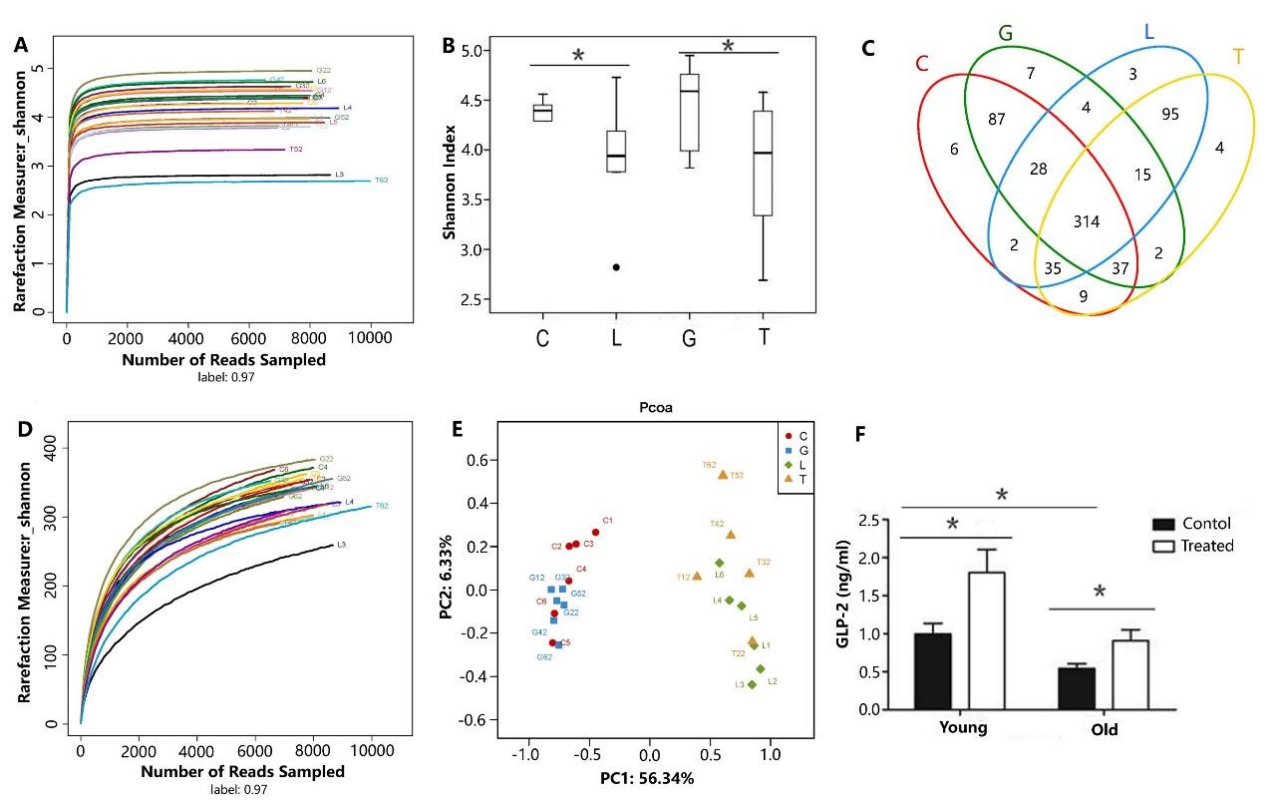
Comparison of diversity indices, Venn diagram, rarefaction curve, PCoA, and plasma concentration of GLP-2 among the 4 groups Comparison of diversity indices including the Shannon Curve (**A**) and Shannon Index (**B**). (**C**) Venn diagram illustrating overlap between the 4 groups. (**D**) Rarefaction curve. (**E**) PCoA was analyzed by unweighted Unifrac. (**F**) Plasma concentration of GLP-2 among the 4 groups. C denotes group C; G denotes group G; L denotes group L; T denotes group T; young denotes group C and group G; old denotes group L and group T; treat denotes treatment with GLP-2; * denotes P<0.05.

The summary information and detailed characteristics of each sample are shown in Table 1. All of the diversity indices (including OTU numbers, Ace index values, Chao index values, and Simpson indices) revealed that gut microbiota diversity was significantly lower in group L than in group C (P < 0.05). Similarly, these indices revealed that gut microbiota diversity was significantly lower in group T than in group G (P < 0.05). However, there was no significant difference in gut microbiota diversity between young rats and aged rats (Fig. 1B, Table 1). The diversity indices revealed that the abundance of intestinal microorganisms in young rats was higher than that in old rats.

Rarefaction curve analysis demonstrated that the curves tended to approach the saturation plateau, while no new OTUs were produced by increasing the number of sequences. This indicated that the sequencing numbers were sufficient (Fig. 1D).

The beta diversity index was calculated to measure the extent of the similarity in the microbial communities and principal coordinate analysis was performed using unweighted UniFrac. Despite significant inter-individual variation, the gut microbiotas of young rats (including rats from group C and group G) and aged rats (including rats from group L and group T) could be divided into two different clusters and separated clearly by principal coordinates analysis (Fig. 1E).

### Bacterial community structure at phylum level

**Figure 2:**
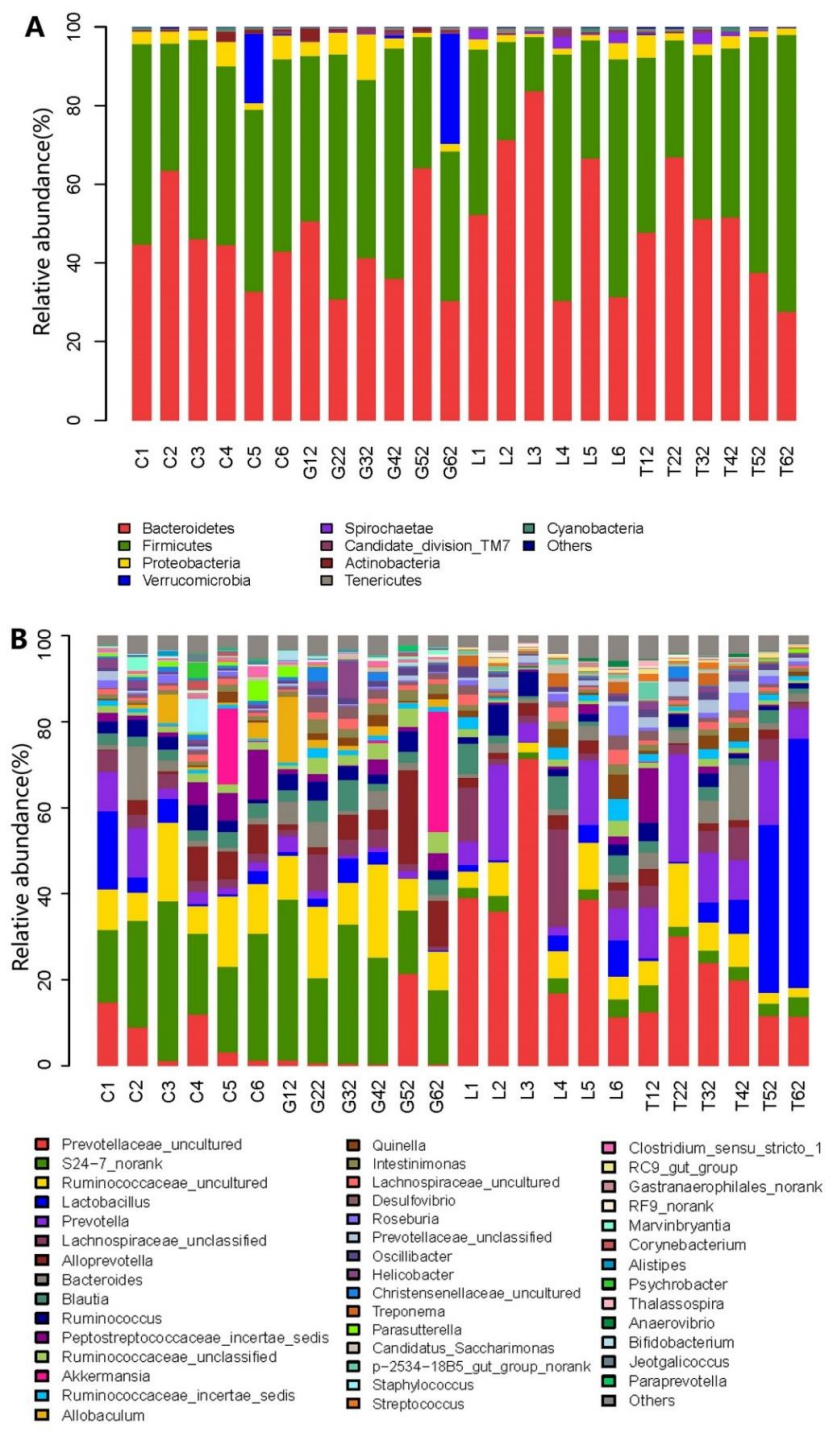
Relative abundance at the phylum level and the genus level in fecal microbiota (**A**) Relative abundance at the phylum level in fecal microbiota. (**B**) Relative abundance at the genus level in fecal microbiota.

Taxonomically, nine different bacterial phyla including *Bacteroidetes*,* Firmicutes*,* Proteobacteria*, *Verruco-microbia*, *Spirochaetae*,* Candidate-divison-TM7*,* Actinobacteria*, *Tenericutes* and *Cyanobacteria* were observed in the fecal microbiota of the samples analyzed (Fig. 2A). Almost all the analyzed sequences (> 95%) belonged to the following five phyla; *Bacteroidetes*,* Firmicutes*,* Proteobacteria*, *Verrucomicrobia* and *Spirochaetae*. The majority of the sequences obtained belonged to *Bacteroidetes* and *Firmicutes*, although the relative abundance of each group differed. The relative abundance of *Firmicutes* was highest in groups C, G and T, while the relative abundance of *Bacteroidetes* was highest in group L.

**Figure 3:**
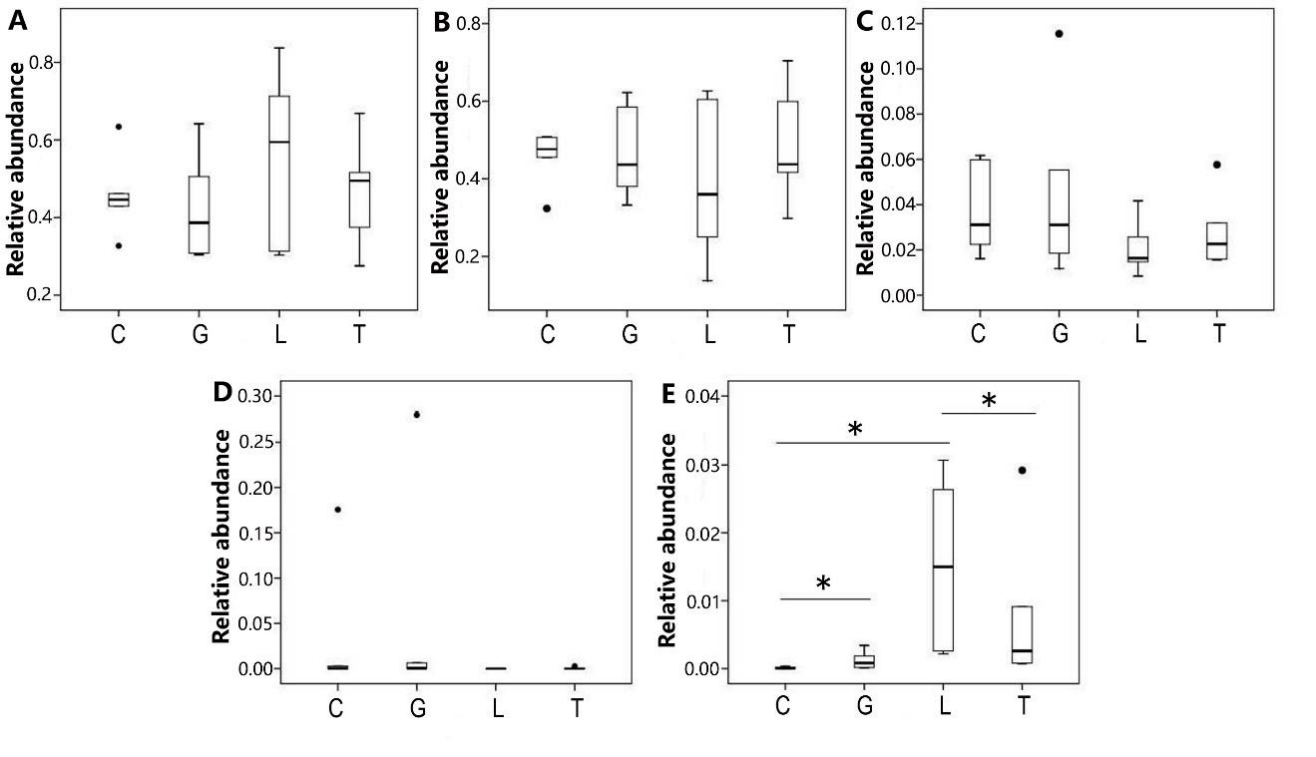
Comparison relative abundance of microbiota at the phylum level among the 4 groups Relative abundance (at the phylum level) of *Bacteroidetes* (**A**), *Firmicutes* (**B**), *Proteobacteria* (**C**), *Verrucomicrobia* (**D**), and *Spirochaetae* (**E**) among the 4 groups. C denotes group C; G denotes group G; L denotes group L; T denotes group T; * denotes P<0.05.

Apart from *Spirochaetae*, no statistically significant difference was observed among the microbial communities of the 4 groups at the phylum level (Fig. 3). The relative abundance of *Spirochaetae* was significantly higher (P < 0.05) in group L than in group C, and significantly lower (P < 0.05) in group T than in group L (Fig. 3E).[Table T2-ad-9-4-566]

**Table 2 T2-ad-9-4-566:** The relative abundance of microbial phyla in the microbiota of Groups C, G, L and T.

Group	*Bacteroidetes*	*Firmicutes*	*Proteobacteria*	*Verrucomicrobia*	*Spirochaetae*
	(%)	(%)	(%)	(%)	(%)
**Group C**	0.457487617	0.45781883	0.037045467	0.029836242	0.000116522
**Group G**	0.422228642	0.465488435	0.043870402	0.047868783	0.001192522
**Group L**	0.559329883	0.389632182	0.020549755	0.000000000	0.015300716
**Group T**	0.470808005	0.482163385	0.027743444	0.0004983	0.007512288

### Bacterial community structure at genus level

At the genus level, *Prevotellaceae_uncultured*,* S24-7_norank*, *Ruminococcaceae_uncultured*,* Lactobacillus*, *Prevotella*, *Lachnospiraceae_unclassified*, *Alloprevotella*,* Bacteroides*, *Blautia*, *Ruminococcus*, *Peptostrepto-coccaceae_incertae_sedis*, *Ruminococcaceae-unclassified*, *Akkermansia*, *Ruminococcaceae_ incertae_ sedis* and *Allobaculum* were the major fecal genera observed in all of the samples analyzed (Fig. 2B).

The 4 groups shared similar intestinal microbiotas at the taxonomic level, although the relative abundance of the constituents varied. To identify the specific bacterial taxa associated with aging, we compared the fecal microbiota of young rats (group C) with aged rats (group L) using the Mann-Whitney U test. The results showed that there were significant differences in relation to the presence of 22 genera between the young and the old groups. The relative abundance of *Anaerovibrio*, *Thalassospira*, *Prevotellaceae_unclassified*,* RC9_gut_group*,* Streptococcus*,* Treponema* and* p-2534-18B5_gut_group_norank* (Table 3) was significantly higher (P < 0.05) in group L than in group C, while the relative abundance of *Alistipes*, *Allobaculum*, *Bifidobacterium*, *Christensenellaceae_uncultured*,* Clostridium_sensu_stricto_1*,* Corynebacterium*,* Helicobacter*, *Jeotgalicoccus*, *Marvinbryantia*,* Para-prevotella*, *Parasutterella*, *Peptococcaceae_uncultured*, *Psychrobacter*, *Ruminococcaceae_uncultured* and* S24-7_norank* (Table 3) was significantly lower (P < 0.05) in group L than in group C.

**Table 3:** The relative abundance of microbial genera in the microbiota of Group C and Group L.

Sequence	Genus	Abundance in Group C (%)	Abundance in Group L (%)	Trends in Group L
1	*Alistipes* *	0.004228	0.000141	↓
2	*Allobaculum* *	0.020827	0.000000	↓
3	*Anaerovibrio* *	0.000000	0.003652	↑
4	*Bifidobacterium* *	0.000574	0.000000	↓
5	*Christensenellaceae_uncultured* *	0.008520	0.001827	↓
6	*Clostridium_sensu_stricto_1* *	0.007729	0.000410	↓
7	*Corynebacterium* *	0.005729	0.000000	↓
8	*Helicobacter* *	0.010086	0.002609	↓
9	*Jeotgalicoccus* *	0.003966	0.000000	↓
10	*Marvinbryantia* *	0.006685	0.000059	↓
11	*Paraprevotella* *	0.001615	0.000000	↓
12	*Parasutterella* *	0.013060	0.000063	↓
13	*Peptococcaceae_uncultured* *	0.002393	0.000247	↓
14	*Prevotellaceae_unclassified* *	0.068351	0.355279	↑
15	*Psychrobacter* *	0.006387	0.000000	↓
16	*RC9_gut_group* *	0.000000	0.005918	↑
17	*Ruminococcaceae_uncultured* *	0.114876	0.060653	↓
18	*S24-7_norank* *	0.244996	0.029386	↓
19	*Streptococcus* *	0.001172	0.003484	↑
20	*Thalassospira* *	0.000341	0.001554	↑
21	*Treponema* *	0.000117	0.015301	↑
22	*p-2534-18B5_gut_group_norank* *	0.000000	0.007516	↑

* denotes significantly different from Group C to Group L, P<0.05

**Figure 4:**
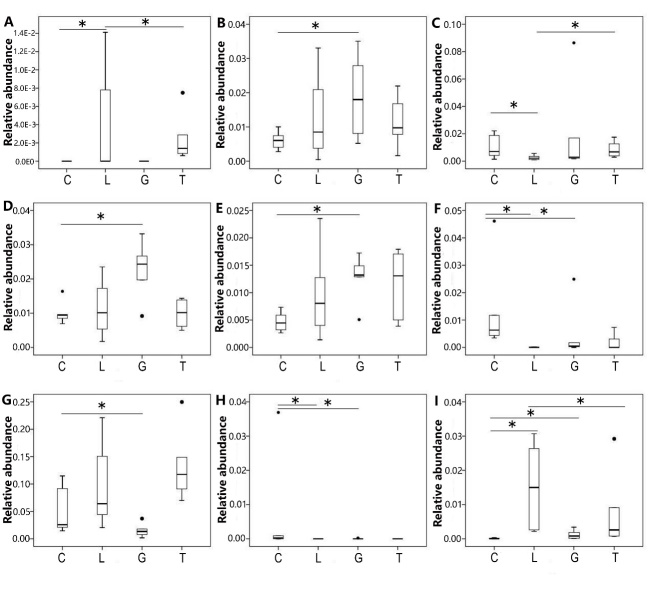
Comparison relative abundance of microbiota at the genus level among the 4 groups Relative abundance (at the genus level) of *Anaerovibrio* (**A**), *Desulfovibrio* (**B**),* Helicobacter* (**C**), *Intestinimonas* (**D**), *Oscillibacter* (**E**), *Parasutterella* (F), *Prevotella* (G), *Psychrobacter* (H), and *Treponema* (I) among the 4 groups. C denotes group C; G denotes group G; L denotes group L; T denotes group T; * denotes P<0.05.

In order to identify if specific bacterial taxa were associated with groups that were administered GLP-2, we compared the fecal microbiota in young rats (groups C and G) and aged rats (groups L and T) using the Mann-Whitney U test. The relative abundance of *Anaerovibrio*, *Desulfovibrio*,* Helicobacter*, *Intestinimonas*, *Oscilli-bacter*, *Parasutterella*, *Prevotella*, *Psychrobacter* and *Treponema* was significantly different (P < 0.05) between the two groups (Fig. 4). Compared to group C, the relative abundance of *Desulfovibrio*, *Intestinimonas*, and *Oscillibacter* was significantly higher (P < 0.05) in group G, while the relative abundance of *Parasutterella*, *Prevotella* and *Psychrobacter* was significantly lower (P < 0.05) in group G. Compared to group L, the relative abundance of *Anaerovibrio* and* Helicobacter* was significantly higher (P < 0.05) in group T. The relative abundance of* Treponem*a was significantly lower (P < 0.05) in group T.

### Plasma concentration of GLP-2

To differentiate the circulating levels of GLP-2 in each group, we compared the plasma concentration of GLP-2 among 4 groups using the ANOVA analysis. We found a significant aging × GLP-2 interaction on plasma concentrations of GLP-2 (Fig. 1F). With aging, the plasma concentration of GLP-2 was significantly lower (P < 0.05) in group L than in group C. With treatment with exogenous GLP-2, the plasma concentration of GLP-2 was significantly higher (P < 0.05) in group G than in group C, the plasma concentration of GLP-2 was significantly higher (P < 0.05) in group T than in group L.

### Correlation between plasma concentration of GLP-2 and gut microbiota

**Figure 5:**
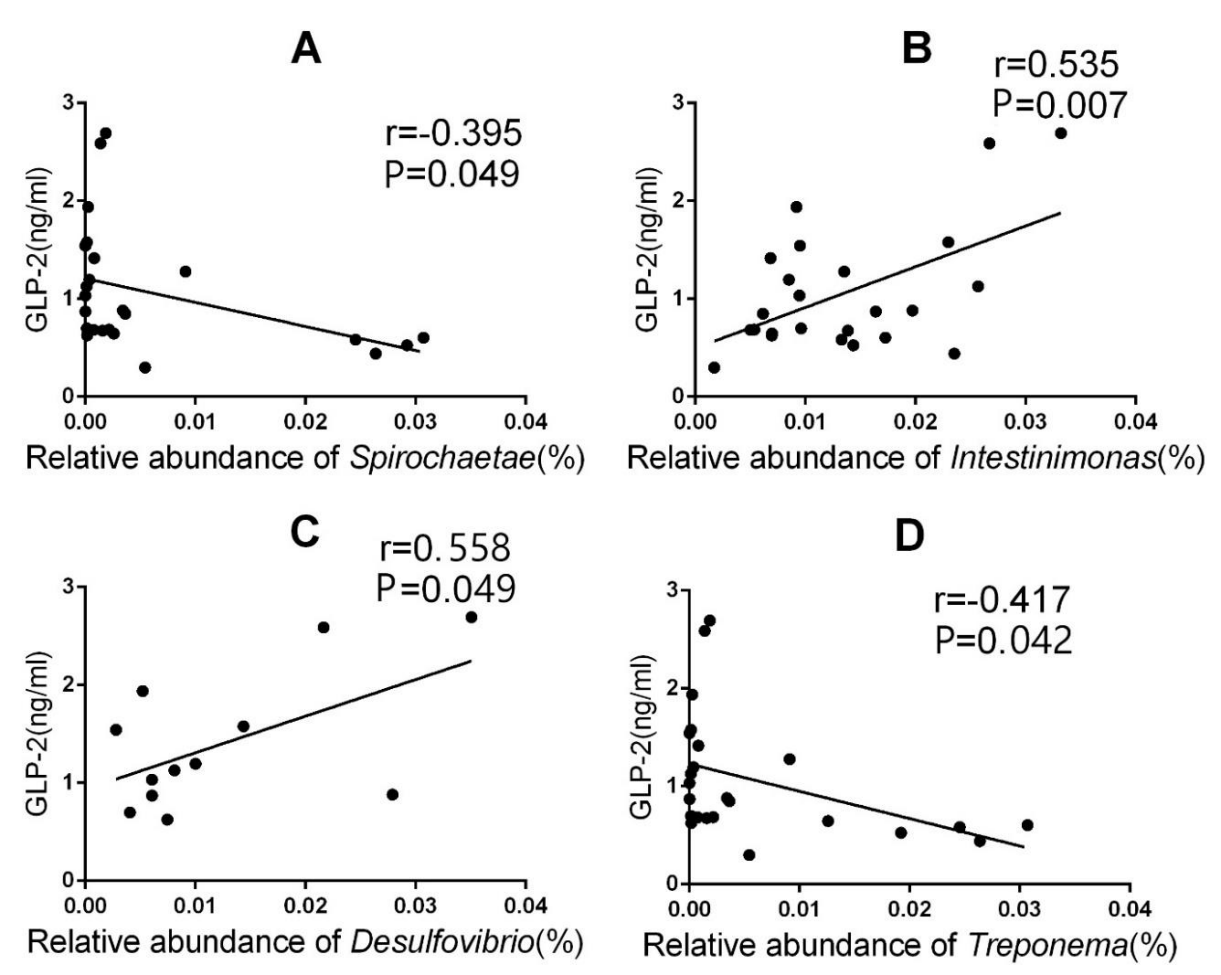
The linear correlation between plasma concentrations of GLP-2 and relative abundance of microbiota The linear correlation between plasma concentrations of GLP-2 and relative abundance of *Spirochaetae* (**A**), *Intestinimonas* (**B**), *Desulfovibrio* (**C**), *Treponema* (**D**).

We analyzed the liner correlation between plasma concentration of GLP-2 and the relation abundance of gut microbiota using Pearson correlation coefficient (r). There was a negative correlation relationship between the plasma concentration of GLP-2 and the relation abundance of *Spirochaetae* (r=–0.395, P<0.05,Fig. 5A), *Treponema* (r=–0.417, P<0.05,Fig. 5D). When the plasma concentration of GLP-2 increased, the relative abundance of *Spirochaetae*, *Treponema* was reduced accordingly. On the other hand, there was a positive correlation relationship between the plasma concentration of GLP-2 and the relation abundance of *Intestinimonas* (r=0.535, P<0.05; Fig. 5B), *Desulfovibrio* (r=558, P<0.05; Fig. 5C). When the plasma concentration of GLP-2 increased, the relative abundance of *Intestinimonas*, *Desulfovibrio* was increased accordingly.

## DISCUSSION

Entering into the 21st century, the life expectancy of humans is rapidly increasing. This dramatic increase in life expectancy has resulted in a concomitant increase in aging-associated diseases. Thus, it is imperative that we investigate the many factors that are believed to underpin aging. One of the factors that has generated a lot of interest in recent years in relation to its role in the aging process is the composition of the intestinal microbiota.

In this current study, we observed significant differences in the diversity and structure of the intestinal microbiota of young and aged rats. The diversity of the intestinal microbiota of rats was significantly reduced in the aged group compared with the young group. This result is consistent with previous reports that demonstrated that aging correlates with a reduction in intestinal microbiota diversity in humans and rats [6, 16]. In this study, we confirmed that aging results in a reduction in the diversity of the intestinal microbiota. Apart from *Spirochaetae*, which was relatively more abundant in rats from group L compared with those from group C, no significant differences were observed at the phylum level in the intestinal microbiota of rats from all groups. A study by Distrutii et al. (2014) suggested that the abundance of *Firmicutes* and *Proteobacteria* was significantly reduced in rats aged 22 months compared with 3-month-old rats. Conversely, the relative abundance of *Bacteroidetes* [17] was greater in 22-month-old Wistar rats than in 3-month-old Wistar rats. It is has been suggested that the proportion of *Bacteroidetes*/*Firmicutes* (B/F) increases with age. However, a number of studies have also demonstrated that the proportion of B/F diminishes upon aging. Thus, we cannot currently draw a conclusion from these results.

At the genus level, *Allobaculum*, *Bifidobacterium* and other beneficial bacteria (in rats) were significantly lower in group L than in group C. Conversely, *Anaerovibrio*, *Thalassospira*, *Streptococcus*, *Treponema* and other pathogenic bacteria or opportunistic pathogens (in rats) were more abundant in group L than in group C. Guigoz et al. suggested that autoimmune tolerance is reduced as a consequence of changes to the intestinal microbiota upon aging. This report also suggested that these changes result in the activation of abnormal immune reactions which can cause intestinal mucous membrane inflammation [18]. An article by Schiffrin et al. demonstrated that aging correlates with an increase in *Enterobacteria* and other gram-negative bacteria in the intestinal tract. The higher prevalence of gram-negative bacteria can lead to an increased release of lipopolysaccharides. Lipopolysaccharides are absorbed into the bloodstream across the gastrointestinal barrier, resulting in endotoxemia and the synthesis of a large number of inflammatory factors [19]. The release of these factors is increasingly important when the barrier function associated with the intestinal mucous membranes is compromised. Aging is also closely associated with chronic low-grade inflammation [20]. As suggested by the results of this current study, aging could result in an obvious increase in enterogenous opportunistic pathogens including *Thalassospira*, *Streptococcus* and *Treponema*. Conversely, the number of beneficial bacteria such as *Allobaculum* and *Bifidobacterium* is significantly reduced upon aging. These alterations to the intestinal microbiota are likely to result in chronic low-grade inflammation.

GLP-2 is a beneficial factor involved in nutrient uptake and barrier protection in intestinal mucous membranes. As a nutritional factor of intestinal epithelial cells, secretion of GLP-2 is reduced with aging in previous studies [21]. In this current study, we observed significant reduce of endogenous secretion of GLP-2 in old rats, which was consistent with previous reports. The circulating concentrations of GLP-2 in young and old rats were increased significantly after intraperitoneal injection of GLP-2 to rats.

A study conducted by Cani et al. suggested that *Bifidobacterium* could increase the production of endogenous GLP-2 in mice. This genus is also involved in improving the intestinal barrier function in diabetic and obese mice [11]. Intestinal pathogens cause chronic low-grade inflammation of the intestinal barrier following age-induced injury. Therefore, the question remains as to whether alterations to the intestinal barrier function affect the composition of the intestinal microbiota. Up until now, very little research has been conducted to investigate this phenomenon. In this current study, we observed that GLP-2 does not significantly affect the diversity of the intestinal microbiota in rats. It is possible that the intestinal microbiota remains relatively constant over time and treatment with GLP-2 for 2 weeks is not enough to facilitate a change in the diversity of the intestinal microbiota. However, we did reveal that GLP-2 does influence the structure of the intestinal microbiota of rats. At the phylum level, the abundance of *Spirochaetae* was significantly reduced in group T compared with group L; while upon aging, there was an increased abundance of *Spirochaetae* in group L compared with group C. Interestingly, treatment with GLP-2 results in a reduction in *Spirochaetae* in aging rats; this result is in contrast to the increase in abundance in *Spirochaetae* caused by aging in non-treated rats. Some members of the genus *Spirochaetae* including *Leptospira*, *Borrelia* and *Treponema* are pathogenic, causing diseases such as syphilis [22], leptospirosis, relapsing fever, dilated cardiomyopathy [23] and gingivitis [24]. Thus, GLP-2 is capable of reducing age-induced increases in the prevalence of some pathogens in rats. At the genus level, the abundance of *Anaerovibrio* and *Helicobacter* was significantly higher in group T than in group L, the abundance of *Treponema* was significantly lower in group T than in group L. *Treponema* is belong to the phylum of *Spirochaetae* which is harmful for rats. GLP-2 is capable of reducing *Treponema* like the pathogens *Spirochaetae* in rats. The harmful bacteria including *Spirochaetae* and *Treponema* was negative correlated with the plasma concentration of GLP-2. Upon aging, the abundance of *Helicobacter* in group L was significantly lower than that in the young rat group. It is possible that GLP-2 administration can cause an increase in *Helicobacter* in aging rats. *Helicobacter* is affiliated to *Proteobacteria*, and several *Helicobacter* strains are associated with gastritis, enteritis, liver cancer and other diseases associated with both humans and animals [25]. Thus, it is possible that aging can reduce the abundance of *Helicobacter*, and GLP-2 could alter the extent of this age-related reduction. *Anaerovibrio* is a kind of bacteria involved in lipid metabolism, including the decomposition of triglycerides to propionic acid, acetic acid and succinic acid [26]. GLP-2 administration could cause an increase in *Anaerovibrio* of aging rats, which is possibly a potentially beneficial bacterium.

In addition, GLP-2 could increase the abundance of beneficial bacteria such as *Desulfovibrio*, *Intestinimonas* and *Oscillibacter* in young rats, while reducing the abundance of opportunistic bacteria such as *Parasutterella*, *Prevotella*, and *Psychrobacter*. The beneficial bacteria including *Desulfovibrio* and *Intestinimonas* was positive correlated with the plasma concentration of GLP-2.

In summary, treatment with GLP-2 appears to be especially beneficial to young rats by a modest increase in the abundance of some beneficial bacteria and a decrease in the abundance of some harmful bacteria. Meanwhile, GLP-2 obviously changes the intestinal microbiota of aging rats by decreasing the abundance of some harmful bacteria and increasing some potentially beneficial bacteria. Therefore, this study confirms that GLP-2 can moderately change the intestinal microbiota of rats. However, further studies are required to more extensively assess the role of GLP-2 during aging.
